# Augmented Reality for Presenting Real-Time Data During Students’ Laboratory Work: Comparing a Head-Mounted Display With a Separate Display

**DOI:** 10.3389/fpsyg.2022.804742

**Published:** 2022-03-07

**Authors:** Michael Thees, Kristin Altmeyer, Sebastian Kapp, Eva Rexigel, Fabian Beil, Pascal Klein, Sarah Malone, Roland Brünken, Jochen Kuhn

**Affiliations:** ^1^Physics Education Research Group, Department of Physics, Technische Universität Kaiserslautern, Kaiserslautern, Germany; ^2^Department of Education, Saarland University, Saarbrücken, Germany; ^3^Physics Education Research Group, Faculty of Physics, Georg-August Universität Göttingen, Göttingen, Germany

**Keywords:** Augmented Reality and education, multimedia learning, cognitive load theory, science education, physics laboratory courses, split-attention effect, spatial contiguity principle, coherence formation

## Abstract

Multimedia learning theories suggest presenting associated pieces of information in spatial and temporal contiguity. New technologies like Augmented Reality allow for realizing these principles in science laboratory courses by presenting virtual real-time information during hands-on experimentation. Spatial integration can be achieved by pinning virtual representations of measurement data to corresponding real components. In the present study, an Augmented Reality-based presentation format was realized via a head-mounted display and contrasted to a separate display, which provided a well-arranged data matrix in spatial distance to the real components and was therefore expected to result in a spatial split-attention effect. Two groups of engineering students (*N* = 107; Augmented Reality vs. separate display) performed six experiments exploring fundamental laws of electric circuits. Cognitive load and conceptual knowledge acquisition were assessed as main outcome variables. In contrast to our hypotheses and previous findings, the Augmented Reality group did not report lower extraneous load and the separate display group showed higher learning gains. The pre- and posttest assessing conceptual knowledge were monitored by eye tracking. Results indicate that the condition affected the visual relevancy of circuit diagrams to final problem completion. The unexpected reverse effects could be traced back to emphasizing coherence formation processes regarding multiple measurements.

## Introduction

In science education, conceptual knowledge is very important for capturing structural connections between subject-specific concepts, principles, and procedures in classrooms (e.g., Vosniadou, 2007; Bennet and Bennet, 2008) and is often facilitated by engaging learners in inquiry processes, such as scientific experimentation. The basic idea of inquiry learning is to trigger learning processes by enabling students to follow (professional) scientific methods and practices (Pedaste et al., 2015). This student-centered perspective demands active knowledge construction by making observations and inferring principles based on gathered information (Lazonder and Harmsen, 2016). Albeit traditional hands-on inquiry-based laboratories allow for unique experiences, pure physical lab work does not ensure positive learning outcomes (Hofstein and Lunetta, 2004; Finkelstein et al., 2005; Wieman and Holmes, 2015; Husnaini and Chen, 2019; Kapici et al., 2019). Successful experimentation demands an adequate level of prior content-related and methodological knowledge or additional instructional support during experimentation (Blanchard et al., 2010; de Jong, 2019). Otherwise, learners might be overstrained by the complexity of the processes and experience cognitive overload situations that hinder learning (e.g., Kirschner et al., 2006).

To compensate for these challenges, hands-on experimentation can be adapted, structured, and augmented by providing supportive virtual information (de Jong, 2019; de Jong et al., 2013), for example by complementing traditional physical manipulatives by virtual representations (Zacharia and de Jong, 2014; Rau, 2020). Displaying virtual information is further known to support transforming conventional learning settings into multimedia settings through the integration of additional external representations into the physical environment (Santos et al., 2014). One technology that has recently moved into the focus of educational research is Augmented Reality (Ibáñez and Delgado-Kloos, 2018; Pellas et al., 2019; Garzón et al., 2020; Bölek et al., 2021; Pathania et al., 2021). This technology enables instructors to integrate virtual information (e.g., measurement data) into the real 3D environment (e.g., experimentation materials) while allowing for interactivity (Milgram and Kishino, 1994; Azuma, 1997; Billinghurst and Dünser, 2012; Radu, 2014; Ibáñez and Delgado-Kloos, 2018). The inherent capabilities to visualize formerly invisible phenomena and abstract quantities (like heat or electricity), spatial and temporal concepts like functional relations between real components and virtual objects meet the demands of supportive educational technology (de Jong et al., 2013; Renkl and Scheiter, 2017), and contribute to the implementation of Augmented Reality in science education (Billinghurst and Dünser, 2012; Akçayır and Akçayır, 2017; Ibáñez and Delgado-Kloos, 2018; Pedaste et al., 2020). Given the possibility of visualizing real-time data and varying the spatial arrangement of information in 3D, AR-supported learning settings can meet design principles derived from established theories of multimedia information processing (Mayer, 2014): especially the principles of spatial and temporal contiguity (Mayer and Fiorella, 2014) can be addressed by the technical options (e.g., Bujak et al., 2013; Radu, 2014; Altmeyer et al., 2020; Garzón et al., 2020; Thees et al., 2020). While meta-analyses have demonstrated that AR has the potential to enhance learning in different content areas (Bacca et al., 2014; Radu, 2014; Limbu et al., 2018; Garzón and Acevedo, 2019; Garzón et al., 2020) and with different instructional methods, such as inquiry-based learning (Garzón et al., 2020; Pedaste et al., 2020), they also indicated a broad variability among the considered studies.

Since comparatively few studies have investigated how particular learning processes can be fostered by applying AR, the present study questions the mutual dependencies between AR-based design and cognitive processes in the context of inquiry-based laboratory scenarios. The overall goal was to verify the superiority of AR-based presentation formats since they allow for spatially integrating real-time measurement data during hands-on investigations. Further, we used eye tracking during conceptual problem-solving tasks before and after the intervention to explore how the way information is presented during learning affects subsequent performance.

The Cognitive Load Theory (Sweller, 2020; CLT; Sweller et al., 1998, 2019) assumes that any kind of learning process burdens the cognitive system and that this burden in turn affects learning success. The CLT differentiates three types of loads that claim resources of the limited working memory capacity: intrinsic cognitive load (ICL; complexity of the information to be processed), extraneous cognitive load (ECL; task-irrelevant cognitive processes), and germane cognitive load (GCL; cognitive resources used to process information into knowledge structures). Referring to the CLT, many guidelines for the design of multimedia instruction suggest minimizing ECL to free up cognitive capacities that could be directed toward ICL and GCL (Mayer et al., 1999; Sweller et al., 2019). In addition, the Cognitive Theory of Multimedia Learning (CTML; Mayer, 2014) emphasizes that meaningful learning requires active engagement in processing the provided multimedia information which is carried out in the verbal and non-verbal channels of the working memory in order to form mental representations of the learning content (Mayer et al., 1999). This process consists of three-steps: selection, organization, and integration. First, a learner selects relevant verbal and visual information. Second, this information is organized in separate verbal and non-verbal channels of the working memory, leading to two distinct mental representations. Finally, these representations are integrated and linked to prior knowledge. The processing of information in both working memory channels leads to more available and elaborated mental models or schemata. Consequently, learners benefit from a simultaneous presentation of words and pictures (multimedia principle; Butcher, 2014).

The CLT and the CTML agree in the assumption that the amount of information that an individual is able to process simultaneously in the working memory is limited. The higher the complexity of integration mechanisms involved in processing of information from multiple sources, the more working memory resources are claimed. Both theories allow for deducing design principles of multimedia instruction that can support cognitive integration processes and for example spare working memory capacity (Mayer et al., 1999; Mayer and Moreno, 2003; Mayer, 2014; Mayer and Fiorella, 2014). Examples are the contiguity principles, which are assumed to reduce extraneous processing and avoid spatial and temporal split-attention effects (Sweller and Chandler, 1994; Mayer and Moreno, 2003; Mayer and Fiorella, 2014; Sweller et al., 2019). The split attention effect can occur in multimedia settings when information that is essential for learning is distributed across different sources (e.g., text and images) and therefore must be mentally integrated by the learners themselves. The effect becomes apparent when the different sources of information provide complex, non-redundant information (Chandler and Sweller, 1991, 1996). The learning environments in the present study were designed according to the principles of temporal and spatial contiguity (Mayer and Moreno, 2003; Mayer and Fiorella, 2014) which state that related verbal and visual information should be provided simultaneously and in spatial proximity to facilitate the integration of information across different representations, and thereby reduce extraneous cognitive load and promote learning outcomes. The positive impact of the spatial contiguity principle on learning is emphasized in the meta-analysis of Schroeder and Cenkci (2018).

Post-test scores and learning gains are considered indicators of successful information processing during multimedia learning. Beyond these product measures, process measures such as eye tracking have proven to be useful methods to investigate further processing steps. The rationale for selecting appropriate metrics of gaze behavior is based on the assumption that during visual tasks, gaze behavior is associated with attention allocation (e.g., eye-mind hypothesis; Just and Carpenter, 1980). A growing number of studies could demonstrate that cognitive activities, such as Mayer’s three processes that are involved in the construction of knowledge from multimedia instruction can be investigated by means of specific eye-tracking metrics that are recorded during the learning phase (e.g., Canham and Hegarty, 2010; Eitel et al., 2013; Jamet, 2014; Chen et al., 2015; Krejtz et al., 2016; Alemdag and Cagiltay, 2018). Moreover, some studies indicate that the design of the learning materials not only has a direct effect on gaze behavior during learning, but also on gaze behavior during subsequent processing of related multimedia problem-solving tasks (Klein et al., 2018, 2019b). However, overall, only a few studies have ever used eye tracking to examine visual attention distribution when students solve complex multimedia science problems (e.g., Cook et al., 2008; Tsai et al., 2012; Klein et al., 2019a).

Although AR has been successfully adopted in several studies to promote inquiry learning (Garzón et al., 2020; Pedaste et al., 2020), only a few of them focused on learning via hands-on scientific laboratory work (Kuhn et al., 2016; Ibáñez and Delgado-Kloos, 2018; Thees et al., 2020) or applied AR during the investigation phase of scientific experimentation (Pedaste et al., 2020). However, it is precisely in such learning environments that AR could offer a significant advantage: the AR-based display of external symbolic representations of measured values into the physical experimental setting creates an integrated presentation format that allows for a maximum of spatial proximity between the virtual representations and real components (Billinghurst and Dünser, 2012; Radu, 2014). Previous research revealed that AR-based learning settings can indeed prevent split-attention effects in university STEM laboratory work (Strzys et al., 2018, 2019; Altmeyer et al., 2020). In their field study integrated in a graded university laboratory course, Thees et al. (2020) found a significant reduction of ECL with a medium effect size in favor of the AR setting compared to experimentation with traditional split-source materials. Altmeyer et al. (2020) investigated whether AR could prevent split-attention effects during lab work in the more interactive and dynamic context of electricity laboratories. They assumed that an integrated presentation format as provided in an AR-condition would minimize learners’ extraneous processing compared to a second condition where the same data was provided as a well-arranged matrix on a separate tablet display. However, contrary to the expectation, the results showed no group-specific reduction of ECL and performance scores indicated only moderately higher learning gains on behalf of the AR-supported setting.

Recent studies indicate a research gap concerning the impact of underlying design factors like the spatial presentation of virtual components on effective and efficient learning. The present study aimed to address this gap by further exploring the assumptions of Altmeyer et al. (2020) and expand their findings by contrasting an AR condition and a separate-display condition that made best use of the respective advantages: instead of tablets, an optical see-through head-mounted display (HMD) was used in the AR condition in order to promote the spatial linking of measured values to the corresponding real components as well as to ensure freehand interaction (Kuhn et al., 2016; Strzys et al., 2019; Thees et al., 2020) and thereby reducing interruptions which accompanied the handling of the tablet-based AR setting. For the separate-display condition, a concise separate display of measured values was provided on a tablet using the livestream of the device’s camera to capture the real-world environment.

With respect to the split-attention effect and the contiguity principles from multimedia learning theories, the integrated presentation format implemented with HMD-based AR in the context of electricity experiments was hypothesized to reduce ECL and thereby enhance learning gains.

*Hypothesis 1: Compared to separate-display-based lab work, HMD-based AR lab work leads to reduced ECL.*

*Hypothesis 2: HMD-based AR lab work yields higher learning gains in topic-related conceptual knowledge than separate-display based lab work.*

Since it is expected that lab work in the different experimental conditions affects group-specific learning processes and outcomes, one can assume that the way of approaching conceptual problem-solving tasks changes depending on the instructional condition. To uncover possible changes in problem-solving processes, we used eye tracking in the pre- and post-test. The corresponding explorative research question therefore reads:

*What are the differences between the separate-display condition and the HMD-based AR condition concerning the development of problem-solving-related gaze behavior from pre-test to post-test?*

## Materials and Methods

### Participants

The sample comprises *N* = 107 German university students whose fields of study were associated with mechanical or bio-chemical engineering. All of them attended the same introductory physics lecture at the time of the intervention. In this university lecture, the students had not yet gone through the subject of electrics. However, this is a topic that is repeatedly taught in school. It could therefore be assumed that the students had enough prior conceptual knowledge and experimental skills to refresh and deepen their previous learning via lab work. Participants were randomly assigned to the AR condition (*N* = 49; 80% male; age: *M* = 19.5; *SD* = 3.0, semester: *M* = 2.0; *SD* = 1.5) and the separate-display condition (*N* = 58; 83% male; age: *M* = 20.5; *SD* = 2.2, semester: *M* = 2.1; *SD* = 1.5)^1^. In return for their participation, the students earned a bonus of 5% regarding the final exam score.

### Materials

#### Laboratory Work Instruction

**FIGURE 1:**
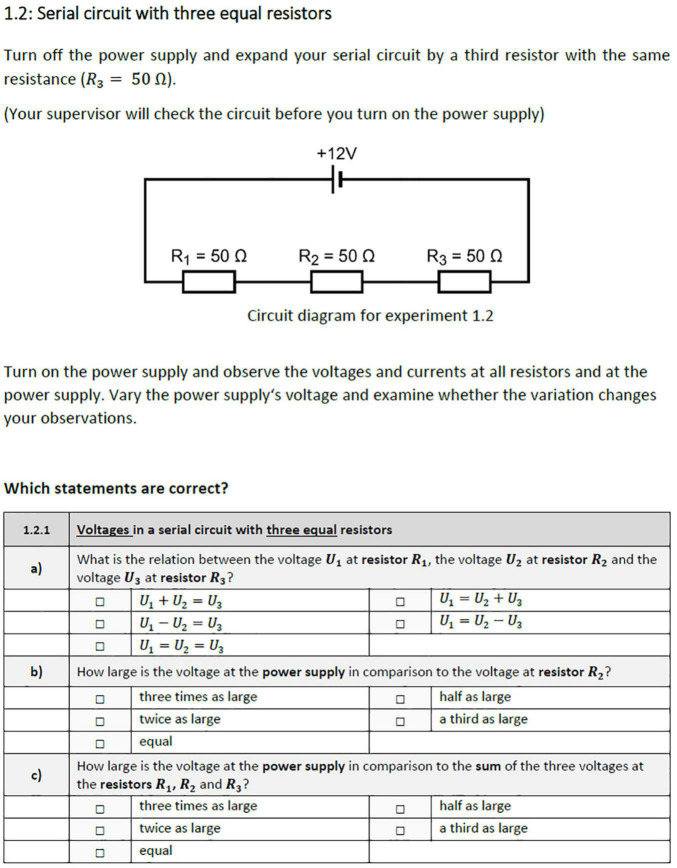
Example of lab work instruction from the work booklet examining a serial circuit consisting of three equal resistors (translated for this manuscript).

Each participant had to conduct six physics experiments following structured task descriptions and corresponding circuit diagrams in a work booklet (Figure 1). Instructions guiding the inquiry process were identical for each task and comprised an explanatory page describing the setup, a schematic circuit diagram, and a set of six single-choice items related to the observations during the experiment. Three of those tasks, respectively, examined serial and parallel circuits with gradually increasing complexity across each set of three tasks. Participants had to set up real circuits successively according to the given circuit diagrams by using a fixed set of components (i.e., a voltage source, cables, and five boxes with resistors). A supervisor corrected the setup if necessary. Participants then observed the behavior of current and voltage at every component while manipulating the source voltage. Corresponding real-time measurement data were presented matching the assigned condition. No further guidance or support was provided. While experimenting, participants completed single-choice items included in the work booklet that dealt with the relation of voltage or amperage at the electronic components of the built-up circuit (Figure 1). The instructions were based on the corresponding introductory physics laboratory courses. Both conditions received the same instructional material.

#### Technology-Enhanced Learning Environment

**FIGURE 2:**
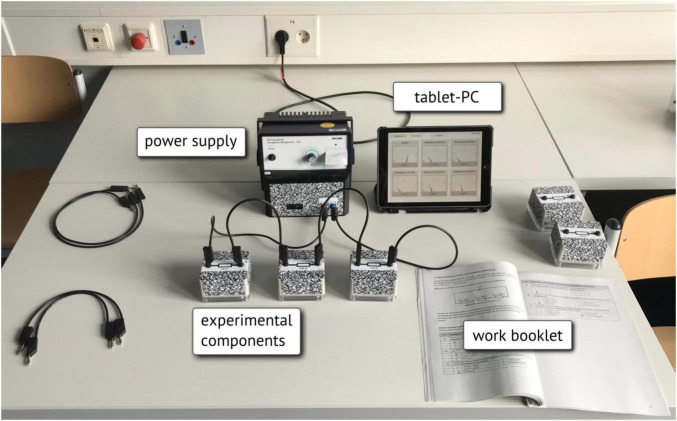
Illustration of the experimental setup with the matrix visualization on a tablet for the separate-display condition.

The learning environment was made up of the work booklet, the experimental components, and a display device (see Figure 2). Traditional electrical components were enhanced by integrating custom-designed measurement nodes. These nodes wirelessly communicated real-time experimental data to a digital assistive system where the data were visualized. A tablet (Apple iPad) was used for the separate-display condition and an optical see-through HMD (Microsoft HoloLens) as the displaying technology for the AR condition. Both conditions were provided with the same representational form inspired by traditional data displays showing a needle deflection for a fixed value range and a numerical value of the raw data (Kapp et al., 2019).

**FIGURE 3:**
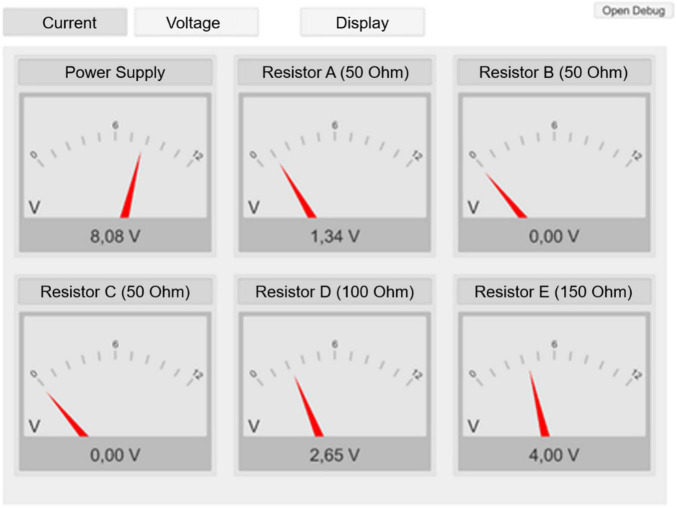
Screenshot of the matrix visualization as presented on the tablet (translated for this manuscript).

For the separate-display condition, the visualization consisted of a well-arranged matrix of the measurement data on the display of the tablet (Figures 2, 3). Participants were able to choose between the presentation of voltage or amperage using dedicated buttons in the application (Figure 2) and via a virtual button in the AR environment.

**FIGURE 4:**
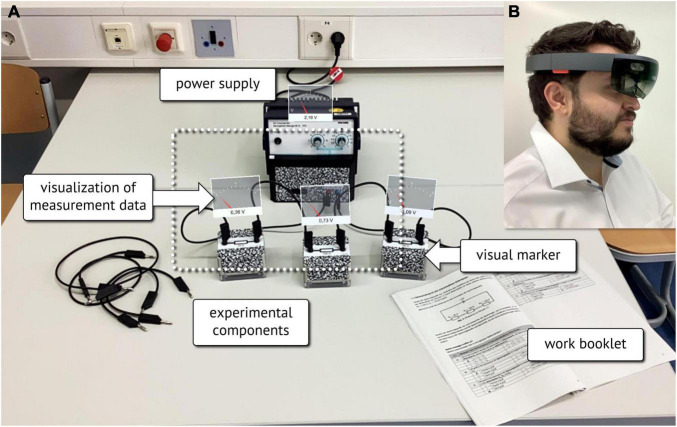
**(A)** Illustration of the AR view as seen through the HoloLens (the white dotted rectangle indicates the limited field of view), **(B)** Researcher wearing a HMD.

For the AR-condition, the stand-alone HMD utilized transparent displays to integrate computer-generated images into the user’s field of view. The visualizations of the measurement data were visually anchored to the relating experimentation components (Figure 4) utilizing an automated recognition of visual markers affixed to the experimental equipment (Figures 2, 4). While sitting in front of the experiment, the small field of view of the HMD limited the number of data displays that could be observed without turning one’s head (Figure 4). This limitation became more critical the more devices and their data displays had to be observed.

For further details on the technological infrastructure, see Kapp et al. (2019) and Altmeyer et al. (2020).

#### Test Instruments

**FIGURE 5:**
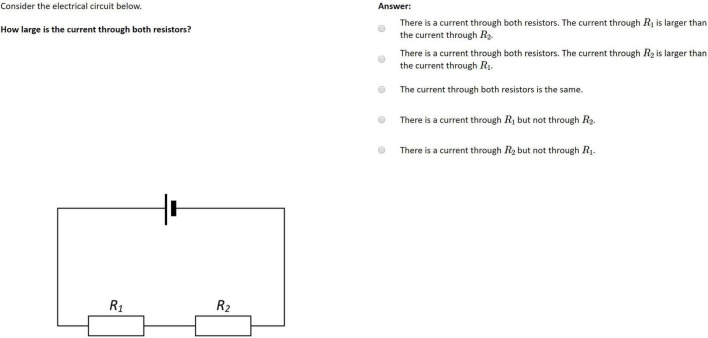
Example of a conceptual knowledge item as presented to the participants (Urban-Woldron and Hopf, 2012; translated for this manuscript).

To evaluate the conceptual understanding of electrical circuits ten items from a power test used by Altmeyer et al. (2020) were applied during both pre- and post-test. The single-choice items were based on the established test by Urban-Woldron and Hopf (2012) and Burde (2018) and examined basic concepts toward parallel and serial electric circuits as well as Kirchhoff’s laws. Each item stem consisted of a brief description of a circuit followed by a question and a corresponding circuit diagram (Figure 5). Distractors represented common misconceptions from basic electrics, such as sequential reasoning and current consumption (Engelhardt, 1997). We adapted the terminology and the symbols used in the circuit diagrams to match the instructions.

As the underlying knowledge test was originally intended to assess a broad variety of different circuits and topic-related physical laws, the applied set of items also covers circuits that were not part of the lab-work phase but demand the application of the same laws and concepts. In this sense, we differentiate between two sets of items for subsuming analyses and discussions: Five items examined circuits that were exact the same as during the lab-work and those items were therefore classified as related to the instruction. The remaining five items referred to circuits that differed significantly from the intervention and were therefore classified as not related to the instruction.

The sequence of the items was identical for every student.

The items of the conceptual knowledge test were presented on a 22-inch computer screen (1920 × 1080 px; refresh rate 75 Hz). Eye movements were recorded with a Tobii X3-120 stationary eye-tracking system to detect fixations and saccades. In order to assign different eye movement types, an I-VT (Identification by Velocity Threshold) algorithm was applied (thresholds: 8500°/s^2^ for the acceleration and 30°/s for the velocity). Prior to presenting the items of the pre- and the post-test, nine-point calibration runs were carried out for each participant to ensure that the eye movement measurements were accurate. After each calibration run, the eye-tracking software provided accuracy and precision measures for quality feedback. If the results were not satisfactory, the calibration was rerun and repeated until both the software and the experimenter were satisfied with the accuracy of the calibration. This calibration procedure worked very well for all subjects in the present study.

**FIGURE 6:**
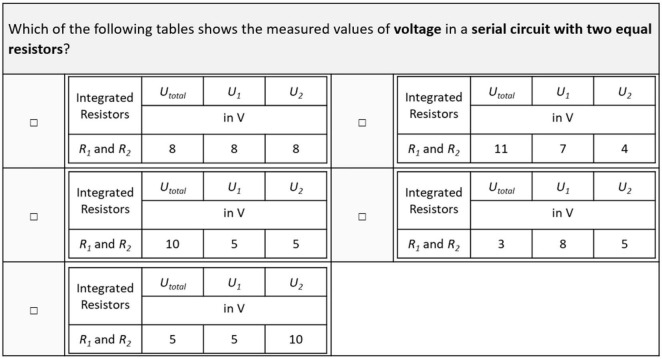
Exemplary item on specific knowledge on the behavior of measurement data (translated for this manuscript).

In addition to the rather general conceptual knowledge test, Altmeyer et al. (2020) presented 12 tasks on measurement data in serial and parallel circuits in their post-test. With respect to the current study, these 12 items were split into two parallelized task sets of comparable difficulty, consisting of six items each. The two sets were used to assess concrete and specific knowledge on the behavior of voltage and amperage in parallel and serial circuits before and after the intervention. Each single-choice item contained a written description of a circuit that had to be matched to one of four tables of measurement data (see Figure 6). Participants had to compare the ratios of the numerical values to the structure of the given circuit. As learners were introduced to these functional relationships during the laboratory work, different value ranges were used in this test to avoid simple recalls of numerical values.

To investigate participants’ cognitive load during the lab-work phase, they were given an adapted version of a seven-point rating scale by Klepsch et al. (2017) during the post-test. We used the second version of their naïve rating scale, where participants rate their agreement with eight statements concerning their cognitive processing but adapted these statements toward our lab-work context. The scale was developed in German and has proven to measure intrinsic, extraneous, and germane cognitive load in various learning situations with an acceptable internal consistency (Cronbach’s alpha: ICL = 0.81, ECL = 0.86, GCL = 0.67; Klepsch et al., 2017). According to the review of Buchner et al. (2022), such a differential measurement of cognitive load has yet been scarcely applied in the context of AR in education.

To investigate the usability of the deployed educational technologies, the System Usability Scale (SUS) by Brooke (1996) was used. Subjects assessed their agreement with 10 items about the handling and usefulness of the specific technological condition on a five-point scale. A German translation of the SUS in which the term “system” was concretized as “interaction between the digital assistive system (tablet or HMD), the corresponding software application, and the experimental equipment” was used (Thees et al., 2020).

Furthermore, the time students needed to conduct each experiment was collected as time-on-task. Additionally, it was noted if students correctly built up the real circuit based on the schematic in their first attempt.

### Research Design

For the present study, a two-group pre-test–post-test design was applied. As a between-subjects factor, the type of spatial presentation of measurement data was varied by randomly assigning the participants to either the AR-supported condition (AR group) with the integration of data visualization and real component or the separate-display condition (separate-display group) with a well-arranged matrix of multiple data visualizations. Materials and procedures were largely based on Altmeyer et al. (2020).

### Procedure

**FIGURE 7:**
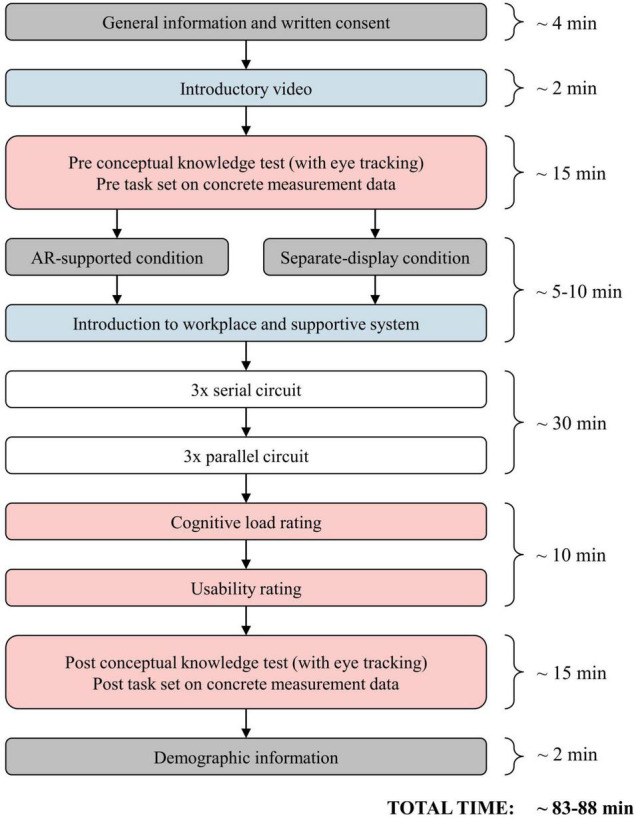
Experimental procedure.

First, participants received general information on the study and data protection and provided written consent for participation. Figure 7 depicts the subsequent experimental procedure.

To begin, participants were presented with a short introductory video on a computer screen that explained the basics of the physical quantities of voltage, amperage, and resistance in order to activate learners’ prior knowledge and to present the terminology used in further instructions. Thereafter, participants completed the pre-test, consisting of a conceptual knowledge test and the first set of tasks on measurement data in different circuits. During the concept test, participants’ eye movements were recorded. Subsequently, students began the lab work by being introduced to their workplace via their work booklet, where all necessary materials to build electric circuits were presented. Participants were assigned to one of the conditions, followed by a first introduction to the tablet or the HMD where they familiarized themselves with the technology (e.g., switching between the displays of voltage and amperage). Participants using the HMD performed a short calibration process to individualize the devices’ optical properties as well as to learn basic gestures to control the device. They were able to keep wearing their own glasses or contact lenses without any limitation. Afterward, students performed six experimental tasks guided by the work booklet. The post-test consisted of a cognitive load questionnaire, a usability questionnaire, the second set of tasks on measurement data, and the repeated conceptual knowledge test, which was also accompanied by eye-tracking measurement. Finally, participants voluntarily provided demographic information (sex, age, course of study).

### Data Coding and Analysis

#### Data on Performance, Cognitive Load and Usability

Mean scores for conceptual knowledge tests, tasks on measurement data, work booklet items, and for each subscale of the cognitive load rating were scaled to [0;1] (low to high). Following Brooke (1996), the score for the usability scale was calculated by multiplying the cumulated item scores (value range: 0 – 4; some items had to be inverted first) with the factor 2.5, resulting in a value range of [0;100] (low to high).

All further analyses considered α = 0.05 as the (global) significance level for type I error. Two-tailed independent-samples *t*-tests were used to analyze differences between groups for those variables that were measured one single time and for which no directed hypothesis was available (cognitive load rating scores for ICL and GCL, work booklet item score, usability score). Concerning ECL rating scores, a one-tailed independent-samples *t*-test was applied to test for differences between the conditions. Since the three facets of cognitive load are considered as related factors (Klepsch et al., 2017), we assumed a case of multiple testing. Therefore, the individual significance level for type I error for each *t*-test was adjusted to α_*adjusted*_ = α/3 ≈0.017 (Bonferroni-Correction).

Further analyses for performance (conceptual knowledge scores, scores from tasks on measurement data) included time as a within-subject factor in addition to the between-subject factor group. Concerning the conceptual knowledge test, a two-factor mixed analysis of variance (ANOVA) was performed to reveal the main effects of group and time toward general topic-related concepts. A subsuming mixed ANOVA included the classification of conceptual knowledge items into related to the instruction and not related as a second within-subject factor in order to explore the necessity of focusing on instruction-related items based on the significance of the triple-interaction group x time x classification. Afterward, instruction-related items were focused and group-specific differences in post-test scores were analyzed via an analysis of covariance (ANCOVA) to control for the effect of the pre-test scores as covariate. Concerning the post-test scores from tasks on measurement data, a similar ANCOVA was conducted including the pre-test scores as covariate.

#### Eye-Tracking Data

Ott et al. (2018) assumed that if individuals are provided with more than one representation for problem solving, the last fixation before finally completing a task indicates which representation is considered to hold the most available information for problem solution. In the present experiment, we used the eye tracking data for the ten pre-test and post-test items on conceptual knowledge to examine which of the two representations—question text or circuit—that were provided for each item, was used more frequently as a final source of information. We could realize this, as each item of the pre- and post-test was displayed full-screen. When participants had selected an alternative from the available set and were confident with their answer, they clicked on a “Next”-button to move to the subsequent item. This click event was recorded in our log-data. Also, all eye tracking events (such as fixations) were included in our log data. Therefore, using the log data for each person, we were able to determine where their last fixation was before they clicked the “Next”-button on a specific item. Across the ten items of the conceptual knowledge test, the number of last fixations on the text and on the circuit schematics was determined for each person.

## Results

Table 1 shows descriptive results for all dependent variables. For all analyses, requirements for conducting ANOVA (independence of samples, normal distribution of residuals, and homogeneity of residuals’ variances) and ANCOVA (homogeneity of variance) were sufficiently fulfilled.

**TABLE 1:** Standardized means (*M*) and standard deviations (*SD*) for dependent variables, separated by AR and separate-display condition.

		AR-supported lab work *N* = 49	Separate-display lab work *N* = 58
Variable		*M* (*SD*)	*M* (*SD*)
Cognitive load rating			
	ICL	0.26 (0.16)	0.26 (0.15)
	ECL	0.21 (0.16)	0.15 (0.14)
	GCL	0.71 (0.16)	0.73 (0.18)
Pre-conceptual knowledge (all items)		0.56 (0.18)	0.55 (0.14)
	Related to instruction	0.55 (0.23)	0.54 (0.21)
	Not related to instruction	0.58 (0.26)	0.57 (0.23)
Post-conceptual knowledge (all items)		0.55 (0.20)	0.53 (0.18)
	Related to instruction	0.62 (0.21)	0.70 (0.19)
	Not related to instruction	0.48 (0.32)	0.36 (0.26)
Tasks on measurement data			
	Pre-task-set	0.46 (0.30)	0.46 (0.29)
	Post-task-set	0.66 (0.30)	0.78 (0.22)
System usability score (max. 100)		75.4 (13.6)	87.7 (10.9)
Work booklet item score		0.91 (0.11)	0.92 (0.08)

### Effects on Cognitive Load

Results from the independent-samples *t*-tests showed no significant group differences for ICL, *t*(100.1) = 0.15, *p* = 0.878, or for GCL, *t*(104.4) = –0.71, *p* = 0.477. This also applies to the one-tailed *t*-test for ECL, *t*(93.1) = 2.10, *p* = 0.038 > α_*adjusted*_ = 0.017. As shown in Table 1, the separate-display condition revealed lower descriptive ECL rating scores than the AR condition, which formally represent a small effect, Cohen’s *d* = –0.4.

### Effects on Performance

First, the full 10-item conceptual knowledge test score was analyzed via a mixed ANOVA using group as a between-subjects factor and time as a within-subject factor. Levene’s test revealed homogeneity of variance across all four groups, *F*(3,210) = 0.82, *p* = 0.484. The analysis showed no significant main effects for group, *F*(1,105) = 0.22, *p* = 0.640, or time, *F*(1,105) = 1.18, *p* = 0.280, and no significant group x time interaction, *F*(1,105) = 0.06, *p* = 0.810.

Afterward, the 10 items were split up into related to the instruction (five items, see section “Test Instruments”) and not related to the instruction (five items, section “Test Instruments”) and this classification was included as a second within-subject factor. Levene’s test showed significant results among all eight groups, *F*(7,420) = 2.82, *p* = 0.007, which is likely for large sample sizes. After considering the variance ratio, Hartley’s *F*_*max*_ = 2.84, which met the range of the corresponding critical value (Field et al., 2012), homogeneity of variance was still assumed for the present large sample. The mixed ANOVA revealed a significant main effect for the classification, *F*(1,105) = 15.45, *p* < 0.001, η_*p*_^2^ = 0.128, as well as significant interaction terms for time x classification, *F*(1,105) = 67.36, *p* < 0.001, η_*p*_^2^ = 0.391, and for group x time x classification, *F*(1,105) = 8.50, *p* = 0.004, η_*p*_^2^ = 0.075. Other main effects for group, *F*(1.105) = 0.22, *p* = 0.640, and time, *F*(1,105) = 1.18, *p* = 0.280 were not significant; the same was true for group x time interaction, *F*(1,105) = 0.06, *p* = 0.810, and group x classification interaction, *F*(1,105) = 3.69, *p* = 0.058.

To determine the effects of time (pre- vs. post) and group only for the instruction-related items, an analysis of covariance (ANCOVA) for the mean of those items was conducted with group as the between-subject factor and the pre-test scores as the covariate. The correlation between pre-test and post-test scores was significant [*r*(104) = 0.34, *p* < 0.001]. Levene’s test revealed homogeneity of variance between the group-specific post-test scores, *F*(1,105) = 0.65, *p* = 0.421. The ANCOVA showed a significant effect of group on the post-test scores after controlling for the effect of the pre-test scores [*F*(1,104) = 5.40, *p* = 0.022, η_*p*_^2^ = 0.049]. The adjusted means of the post-test scores revealed a small effect size of Cohen’s *d* = 0.31 in favor of the separate-display condition.

To determine the effects of time and condition on the tasks on measurement data, an ANCOVA was conducted with group as the between-subjects factor and the pre-test scores as the covariate. There, Levene’s test also showed significant results for group-specific post-test scores, *F*(1,105) = 5.60, *p* = 0.020. However, homogeneity of variance was still assumed after considering the variance ratio, Hartley’s *F*_*max*_ = 1.86 which was also in the range of the corresponding critical value (Field et al., 2012). The correlation between pre-test and post-test scores was significant [*r*(105) = 0.32, *p* < 0.001]. Consequently, the ANCOVA showed a significant effect of group on the post-test scores after controlling for the effect of the pre-test, *F*(1,104) = 5.66, *p* = 0.020, η_*p*_^2^ = 0.052 in favor of the separate-display condition with a small effect size, Cohen’s *d* = 0.31.

Concerning the lab-work phase, both conditions showed high mean scores for work booklet items (see Table 1). No significant differences were found after applying an independent-samples *t*-test to contrast the two groups, *t*(84.1) = –0.97, *p* = 0.33.

### Usability

Table 1 shows the average usability scores calculated according to Brooke (1996). A *t*-test for independent samples revealed a significant difference between the two conditions, *t*(91.4) = 5.1, *p* < 0.001. Following Bangor et al. (2009), the usability of the separate-display condition can be described as “excellent,” representing the second-best usability level. The usability rating of the AR condition can be classified as “good” which corresponds to the third-best possible level.

### Eye-Tracking Measures of Conceptual Knowledge

Table 2 shows descriptive group-specific results for eye-tracking measures.

**TABLE 2:** Means (*M*) and standard deviations (*SD*) for the eye-tracking measures separated for time (pre- vs. post-test) and group (AR vs. separate-display condition).

	*M* (*SD*)			
	Pre-test		Post-test	
Variable	separate-display	AR	separate-display	AR
Number of last fixations				
Question AOI	1.73 (1.19)	2.08 (1.67)	2.36 (1.43)	1.87 (1.52)
Circuit AOI	5.24 (1.23)	4.88 (1.68)	4.44 (1.51)	4.85 (1.47)

Last fixations on the representations used for problem solving (question text and circuit) were examined. Taking a closer look at the dependent variable last fixations on the question, a mixed ANOVA including group as between-subject factor and time as within-subjects factor revealed no main effect for time or group but a significant interaction between the factors: Only the separate-display condition showed an increase in the number of last fixations on the question from pre- to post-test, *F*(1,105) = 8.31, *p* = 0.005, η_*p*_^2^ = 0.073.

Concerning the number of last fixations on the circuit, the corresponding mixed ANOVA revealed no main effect for group but a main effect for time. Subjects showed fewer last fixations on the circuits of the post- than the pre-test. Furthermore, time significantly interacted with group. This effect indicates that only the separate-display condition declined in their number of last fixations on the circuit from pre- to post-test, *F*(1,105) = 14.29, *p* < 0.001, η_*p*_^2^ = 0.120. Corresponding numerical values are displayed in Table 3.

**TABLE 3:** Results of mixed ANOVAs for the dependent measures of gaze behavior, separated for the effects for the factors time, group and the interaction between time and group (t × g).

Dependent variable	*df* (conditions/error)	*F*			*p*			η_*p*_^2^		
				
		Time	Group	t × g	Time	Group	t × g	Time	Group	t × g
*Number of last fixations*										
Question AOI	1/105	1.80	0.10	7.17	0.183	0.751	0.009**			0.064
Circuit AOI	1/105	7.63	0.01	6.29	0.007**	0.905	0.014*	0.068		0.057

Significance levels for group differences: *p < 0.05, **p < 0.01.

## Discussion

The purpose of the current study was to contrast two different technology-enhanced presentation formats to support traditional physics laboratory experiments examining electric circuits in a structured inquiry format. Based on CLT and CTML, the AR condition was expected to create enhanced spatial contiguity compared to the separate display condition. However, contrary to the expectations, the conditions did not differ regarding ECL. Moreover, the separate-display condition outperformed the AR condition in terms of conceptual knowledge items related to the instruction as well as concerning the tasks on concrete measurement data. Overall, the results challenge the broad evidence for superiority of AR.

### Cognitive Load and Performance

#### Reverse Results

Concerning the experimental task of exploring relationships between physical quantities during an inquiry-based physics experiment, the AR-based integration of the real-world circuit components and their related virtual data displays corresponds to the spatial contiguity principle. Thus, we assumed higher spatial contiguity for the AR condition, which according to theoretical assumptions (e.g., Mayer and Fiorella, 2014) and recent reviews (Schroeder and Cenkci, 2018), was expected to result in a reduction of extraneous processing and higher learning gains. However, none of the hypotheses were supported by our data as the AR condition did not trigger lower ECL ratings or higher learning gains. In fact, the results indicate reverse effects: participants in the separate-display condition achieved significantly higher learning gains regarding conceptual knowledge and performance in the tasks on measurement data, each with small effect sizes. Even for subjective ECL ratings, the descriptive results reveal a tendency in favor of the separate display. In sum, these unexpected, yet consistent results of cognitive load and performance measures strongly indicate the efficacy of a presentation format that was hypothesized to be inferior to the AR condition. These findings contrast both the well-known effects of the spatial contiguity principle (Schroeder and Cenkci, 2018) as well as the pronounced advantages of AR learning (Kuhn et al., 2016; Garzón and Acevedo, 2019; Altmeyer et al., 2020; Garzón et al., 2020; Thees et al., 2020).

In the first place, these findings are quite surprising since the study and its hypotheses were based on the study of Altmeyer et al. (2020) and the fact that the AR-based learning environment corresponds to the principles of spatial and temporal contiguity as well as the overarching goal of AR-based learning to combine real and virtual objects.

While Altmeyer et al. (2020) also could not support the hypothesis of a reduction of ECL for the AR condition in their study, the present findings even point toward opposite effects in favor of the separate-display condition. The main difference between the present study and the one of Altmeyer et al. (2020) were the applied AR-devices (HMD instead of tablets). Accordingly, one might reason that the choice of device could be responsible for whether AR is beneficial or detrimental to learning. However, similar studies comparing HMD-based AR with separate displays in laboratory work have found no negative effects on learning, ECL, or usability (Kapp et al., 2020; Thees et al., 2020). Therefore, it is unlikely that the reverse effects found in the present study can be attributed to the choice of device in general but might stem from a mismatch of the affordances of the chosen device and the cognitive processes that were intended to be triggered in this study to enable successful learning. Thus, we reanalyzed the theory-based idea of an integrated presentation format with respect to an experiment-based learning environment.

#### Theoretical Implications

Some constraints can be derived from a more differentiated look on coherence formation processes of multimedia learning. Depending on the internal structure of the content and the tasks, learners have to perform local and/or global coherence formation processes (Seufert and Brünken, 2004, 2006) to successfully integrate the presented information. According to the authors, the integration of multiple representations is preceded by the understanding of each of the presented single representations by building a coherent mental representation. If this local coherence formation was successful, referential connections between different representations can form an integrated mental representation. This process is called global coherence formation. Searching for referential connections between representations is a demanding process, imposing cognitive load. By successfully interrelating relevant concepts of multiple representations on a deep structural level, learners profit from the formation of global coherence, resulting in the construction of a coherent, elaborated knowledge structure. In this sense, a careful analysis of the learning task should be carried out to assess whether it is more relevant for mental model construction to relate the information within one representation (local coherence formation) or across representations (global coherence formation).

Since an electric circuit is a global system, each manipulation requires taking several measurements to explore mutual interdependencies of the physical quantities and infer underlying principles and concepts (Stetzer et al., 2013). Hence, in the present study, successful local coherence formation by retrieving and comparing measured values is considered crucial for the experimental tasks. While displaying the measured values in close spatial proximity to their circuit components might have facilitated structure-mapping processes between measurement data and experimental set up, the AR condition also resulted in all measurement data being spread across the circuit, which might have made it difficult to relate multiple measurements and form local coherence. This can be interpreted as a case of spatial contiguity failure (e.g., Beege et al., 2019; Cammeraat et al., 2020). In contrast, the measurement matrix on a separate display provided no spatial information but simplified referential connections between single measurement values. Therefore, learners were able to compare several values without performing resource-consuming search processes and they were supported in terms of local coherence formation processes. Thus, even though AR can be used to promote coherence processes in general, it is only helpful if the fostered coherence is beneficial for a specific task. These boundary conditions might have come into effect regarding the results of the present study which therefore opposes the presumed overall superiority of combining real and virtual elements. Consequently, the idea of achieving spatial contiguity in lab work contexts has to be refined.

Although the HMD used in this study enabled hands-free experiences, the benefits of this particular function were not decisive. Rather, it appears that promoting local coherence within measurement data, as achieved by the separate-display condition, was more significant for the present learning content. Moreover, the findings indicate that the HMD’s limited field of view increased the obstruction of the mapping process between single measured values: Participants had to actively engage in turning their view to observe data excluded from their current view and to integrate this information. Therefore, the limited field of view of the HMD might have made it more difficult to observe and compare the dynamic measurement values for the different components. This may explain why the present research could not replicate the results of a very similar study by Altmeyer et al. (2020). The authors did find an advantage of AR over a separate-display condition; however, the AR-experience was realized via a tablet, which enabled the learners to observe all components and their measurement values, possibly facilitating both local and global coherence. However, for a different learning content it might be even helpful to limit the field of view to the most important areas of a setting. Moreover, since latest HMD are constantly improving their field of view, this limitation could soon be overcome by technical advances.

Considering this expanded perspective, the present study contrasted two assistive systems that are intended to foster global coherence formation (AR condition) or local coherence formation (separate-display condition). For the given scientific context and the specific set of tasks, the separate-display condition seems to be the more adequate presentation format to reduce split-attention effects and to foster learning.

#### Eye-Tracking Measures

Eye-tracking data analyses were applied to explore group-specific changes concerning conceptual problem solving from pre- to post-test. The analysis of the eye-tracking data revealed that, in contrast to the AR condition, students in the separate-display group changed their visual behavior before moving on to the next problem from the pre- to the post-test. In the post-test they displayed more last fixations on the questions and fewer last fixations on the symbolic circuits than before the treatment, whereas the AR group did not change regarding these parameters. The type of measurement display used in the learning phase seems to have affected how participants solved similar problems in the subsequent test phase. Following Ott et al. (2018), the symbolic circuits might have become less relevant for the final problem solution in the separate-display group. This result might also reflect the participants’ behavior during experimentation. Probably, the participants in the separate-display condition assigned less of their visual attention to the built-up circuits during the experiments and concentrated more on the displayed measurements on the tablet performing local coherence formation. In order to verify this assumption, future research should also include mobile eye tracking during lab work to compare gaze behavior in different conditions and match gaze behavior in the learning and test phases.

### Limitations

One limitation of the present study is that significant group-specific learning effects were only found for the tasks explicitly related to the concepts that were relevant during the lab-work phase. However, no significant learning effects were found for the full 10-item conceptual knowledge test. This means that the laboratory work probably did not promote the acquisition of the entire scientific concepts. Prior research has shown that it is crucial for conceptual learning that topic-related misconceptions are addressed properly during interventions (e.g., Leinonen et al., 2013). Because the experimental tasks of this study were limited to simple parallel and serial circuits, some of those misconceptions might have been activated, consequently resulting in the wrong answer for corresponding items. Learners might have needed more time or multiple experimentation events to build up knowledge structures that allow for transferring their findings to new circuits.

A further limitation is that the lab work and tasks of the work booklet were rather simple for the students: low cognitive load was reported and high scores for the experimental tasks were achieved. Yet, the positive impact of the application of multimedia design principles like spatial contiguity can only come into full effect for properly challenging tasks (Sweller, 2010).

A third limitation of this study arises from the fact that the effects found cannot be attributed unambiguously to the differences across the two conditions in promoting coherence-formation processes. Especially with regard to the usability of the HMD, there are further issues that could have hindered learning with AR. The usability was not rated as poor in the HMD group, but less good than in the separate display condition. It is possible that the presentation of measured values above the circuit components was very unfamiliar to the learners, while the separate display condition was more in line with the traditional way that students are used to experimenting. However, there are different ways to design student experiments on electrics (e.g., sequential measurements, multiple measurement devices, different types of displays) and we did not inquire in this study what participants’ exact experiences with these were. Furthermore, it is possible that participants were already more accustomed to using and interacting with tablets in general than they were to wearing an HMD. Furthermore, it should be noted that although we have tried to make the representations of the measured values in the two display conditions as equivalent as possible, the two devices do differ in some technical details that may have influenced the learning processes as well (e.g., display resolution, small latency differences).

### Future Directions

Taking a closer look at coherence formation processes during experimentation, future studies should investigate ways to support mapping processes between representations and within the single representation, such as the signaling of referential connections (e.g., Seufert and Brünken, 2004). However, different experimental domains and setups might pose different demands on coherence formation processes. Regarding a learning environment in which the formation of global coherence is especially challenging and relevant, the integrated visualization through a HMD might foster the learning process. This assumption should be investigated by creating an instruction that necessarily requires learners to make referential connections between the experimental environment and the data to successfully solve a task. From a methodological point of view, measuring coherence formation processes could be achieved by applying mobile eye tracking during experimentation, which is available for HMD-based AR-settings since recently (e.g., Kapp et al., 2021).

As mentioned above, the two devices used in the present study differed in some ways that might have been less associated with coherence formation, but still might have influenced learning processes (e.g., usability, familiarity). Future research should take even more care to control for these or keep them constant across the conditions to be compared.

Another important aspect is that research has shown that in both inquiry learning and multimedia learning, the effectiveness of instructional support and the use of design principles are related to the learners’ level of prior knowledge (e.g., Kalyuga, 2007; Schneider and Preckel, 2017). Future research should therefore use sensitive prior knowledge tests and large diverse samples to investigate possible moderating effects of prior knowledge on the effectiveness of AR support for coherence-building processes.

## Conclusion

In the present study, we achieved the implementation of an AR-based learning scenario in the context of science experiments which fulfills the demands of spatial and temporal contiguity by integrating virtual elements, i.e., representations of real-time measurement data, into the real learning environment. Although this combination of real and virtual components is broadly considered as one of the main advantages of AR and the contiguity principles are well-known to foster learning as well as to reduce extraneous load, the AR-based learning environment could not be confirmed as more effective. In contrast, the descriptive results even indicate that the separate-display condition might outperform the AR condition concerning learning gains and cognitive load. This leads to the theoretical assumption of underlying dependencies between the original instruction and the presentation format of the learning-relevant information that outweigh the importance of a simple spatial integration of virtual and real objects.

We therefore suggest including the perspective of coherence formation which allows to focus on mutual dependencies between different information sources and distinguishes between different integration processes. Regarding the current study, the promotion of correspondences within one representation, namely the measurement data, outperformed the support of a global coherence formation. This might be due to the specific and challenging learning tasks that, above all, required subjects to interrelate measured values. The integration of data and physical environment seemed to be of less importance.

Furthermore, analyses of the learners’ visual behavior indicate a relationship between the presentation format during the experimentation and processing of subsequent conceptual knowledge items. In this sense, eye tracking also appears to be a promising approach to analyze the impact of coherence formation processes during the learning acquisition phase in future studies.

In accordance with multimedia learning theories, the present study still supports the idea of digital assistance in science laboratory learning environments. Our findings highlight the key role of the original instruction as a starting point for designing learning scenarios. Hence, we want to encourage researchers and instructors to first thoroughly consider what kinds of coherence-formation processes the learning task requires, and to precisely adapt the choice of technological support to it.

## Data Availability Statement

The raw data supporting the conclusions of this article will be made available by the authors, without undue reservation.

## Ethics Statement

Ethical review and approval was not required for the study on human participants in accordance with the local legislation and institutional requirements. The patients/participants provided their written informed consent to participate in this study. Written informed consent was obtained from the individual(s) for the publication of any potentially identifiable images or data included in this article.

## Author Contributions

MT: conceptualization, methodology, formal analysis, investigation, writing – original draft, writing – review and editing, visualization, and supervision. KA: methodology, formal analysis, investigation, writing – original draft, and writing – review and editing. SK: methodology, formal analysis, software, investigation, writing – review and editing, and visualization. ER: formal analysis, investigation, writing – original draft, and visualization. FB: investigation, writing – review and editing, and visualization. PK: methodology, formal analysis, resources, writing – original draft, and project administration. SM: conceptualization, methodology, formal analysis, investigation, writing – original draft, and writing – review and editing. RB: conceptualization, resources, and funding acquisition. JK: conceptualization, resources, writing – original draft, writing – review and editing, project administration, and funding acquisition. All authors contributed to the article and approved the submitted version.

## Conflict of Interest

The authors declare that the research was conducted in the absence of any commercial or financial relationships that could be construed as a potential conflict of interest.

## Publisher’s Note

All claims expressed in this article are solely those of the authors and do not necessarily represent those of their affiliated organizations, or those of the publisher, the editors and the reviewers. Any product that may be evaluated in this article, or claim that may be made by its manufacturer, is not guaranteed or endorsed by the publisher.

## Footnotes

1 The authors used a subsample of the dataset in a further publication (Thees et al., 2021) for analyzing the validity of cognitive load scales. There, a second cognitive load rating scale based on Leppink et al. (2013), was reported which was surveyed during the study. For the present analyses, we considered the scale that was slightly superior regarding its sensitivity toward ECL in terms of validity based on content.
